# The m^6^A writer VIRMA regulates the developmental elimination of retinal astrocytes and retinal vascular integrity maintenance

**DOI:** 10.4103/NRR.NRR-D-25-00295

**Published:** 2026-01-27

**Authors:** Cuiting Wu, Xiaoli Wu, Min Wu, Xidan Zhou, Jie Tu, Bin Shen, Tao Zhou

**Affiliations:** 1 Shenzhen Neher Neural Plasticity Laboratory, Shenzhen Key Laboratory of Drug Addiction, the Brain Cognition and Brain Disease Institute, Shenzhen Institutes of Advanced Technology, Chinese Academy of Sciences, Shenzhen, Guangdong Province, China; 2 University of Chinese Academy of Sciences, Beijing, China; 3 Shenzhen-Hong Kong Institute of Brain Science-Shenzhen Fundamental Research Institutions, Shenzhen, Guangdong Province, China; 4 Faculty of Life and Health Sciences, Shenzhen University of Advanced Technology, Shenzhen, Guangdong Province, China; 5 State Key Laboratory of Reproductive Medicine and Offspring Health, Women’s Hospital of Nanjing Medical University, Nanjing Maternity and Child Health Care Hospital, Gusu School, Nanjing Medical University, Nanjing, Jiangsu Province, China

**Keywords:** astrocyte elimination, development, N^6^-methyladenosine (m^6^A), microglial phagocytosis, retina, single-nucleus RNA sequencing, vessel regression, VIR-like m^6^A methyltransferase associated (VIRMA), visual impairment

## Abstract

Proper migration and formation of the astrocyte network are essential for retinal vasculature and visual function. However, the underlying mechanisms remain incompletely understood. In the present study, we identified protein virilizer homolog VIR-like m^6^A methyltransferase associated (VIRMA)—the core scaffolding protein of the N^6^-methyladenosine (m^6^A) methyltransferase complex—as a novel regulator of retinal astrocytes and visual function. We demonstrated that the conditional knockout of VIRMA in retinal astrocytes impaired microglia-mediated phagocytic clearance, leading to abnormal astrocyte accumulation during postnatal development. Notably, this disruption in cellular homeostasis preceded subsequent pathological vascular remodeling. Although initial retinal vascularization appeared normal in VIRMA-deficient mice, persistent vascular plexus instability developed, ultimately leading to vessel regression and irreversible visual impairment. Mechanistically, single-nucleus transcriptomic analysis revealed that the loss of VIRMA disrupted the expression of multiple m^6^A-modified genes that are involved in extracellular matrix organization and angiogenesis. These molecular changes were correlated with impaired astrocyte–endothelial cell communication and contributed to the breakdown of vascular homeostasis. Collectively, our study identifies VIRMA/m^6^A as a novel regulator of the developmental elimination of retinal astrocytes and the maintenance of neurovascular integrity. This research provides new insights into astrocyte-related retinopathies and highlights potential therapeutic targets for vascular-associated visual disorders.

## Graphical abstract

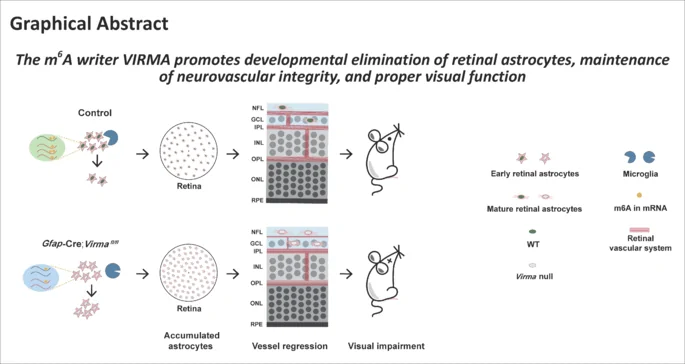

## Introduction

Astrocytes serve critical roles in the establishment of the blood–brain barrier and blood–retina barrier (BRB) as well as in synaptic pruning and neuronal communication (Kettenmann et al., 2005; Volterra and Meldolesi, 2005; Stevens et al., 2007; Allaman et al., 2011). In the retina, astrocytes play an important role in vascularization, followed by vessel remodeling and regression (Dorrell et al., 2010; Korn and Augustin, 2015; O’Sullivan et al., 2017). They originate in the optic nerve head and subsequently migrate to the nerve fiber and ganglion cell layers. During the migration process, astrocytes function as a scaffold for blood vessels to follow and cross the inner surface of the retina via the secretion of growth factors such as vascular endothelial growth factor (VEGF) (Stone et al., 1995; Dorrell et al., 2002). Post-migration, the star-shaped astrocytes form a web-like network within the nerve fiber layer, which is essential for preserving endothelial tight junction integrity and BRB functionality (Ridet et al., 1997). Numerous retinal disorders, arising from disruptions in microvascular remodeling or pathological vessel pruning and regression, may ultimately result in irreversible blindness (Korn and Augustin, 2015), including the early stage of diabetic retinopathy (non-proliferative diabetic retinopathy), familial exudative vitreoretinopathy, hypertensive retinopathy, or rare idiopathic diseases (e.g., retinal vasculitis) (Gilmour, 2015; Lim et al., 2019; Hua et al., 2020; Maurissen et al., 2024; Stuebiger et al., 2024). Across these conditions, experimental studies consistently demonstrate pathological changes in astrocytes accompanied by microvascular dysregulation (Yun et al., 2016; Matteucci et al., 2019; Yang et al., 2022a; Dharmarajan et al., 2023). However, the underlying mechanisms have not yet been fully addressed.

The epitranscriptomic regulator N^6^-methyladenosine (m^6^A) has recently been implicated in central nervous system development and neurodegenerative pathologies (Fu et al., 2014; Livneh et al., 2020; Boulias and Greer, 2023; McMillan et al., 2023; Wang et al., 2023b; Liufu et al., 2024; Li et al., 2025). m^6^A is the most prevalent internal modification of mammalian mRNA (Dominissini et al., 2012; Shi et al., 2019); it orchestrates the metabolism of transcripts, including stability, nuclear export, alternative splicing, translation, and degradation (Jiang et al., 2021). m^6^A is deposited on newly synthesized transcripts in the nucleus by a multicomponent methyltransferase complex (m^6^A writer), which consists of the catalytic subunit m^6^A–METTL complex (MAC) and the regulatory subunit m^6^A–METTL-associated complex (MACOM) (Knuckles et al., 2018). N(6)-adenosine-methyltransferase METTL3 and METTL14 form the core catalytic subunit MAC, and pre-mRNA-splicing regulator WTAP, VIR-like m^6^A methyltransferase associated (VIRMA), zinc finger CCCH domain-containing protein 13, RNA binding motif protein 15/15 paralog, and E3 ubiquitin-protein ligase Hakai form the regulatory subunit MACOM. VIRMA is the core structure of MACOM. It is speculated that MACOM binds its RNA substrate via the N- and C-terminal of VIRMA, thus enhancing the MAC binding affinity of the RNA substrate; this indicates the pivotal role of VIRMA in helping MACOM to assemble and interact with MAC to form an active m^6^A writer complex (Su et al., 2022).

In the retina, m^6^A governs retinal neuron survival via METTL14 and YTH domain-containing family protein 2 (Niu et al., 2022; Yang et al., 2022b), cell cycle progression and cell fate definition of retinal progenitor cells via METTL14 and METTL3 (Xin et al., 2022; Li et al., 2023), and ocular angiogenesis (including retinal, choroidal, corneal neovascularization) via METTL3 and alpha-ketoglutarate-dependent dioxygenase FTO (Shan et al., 2020; Yao et al., 2020; Wang et al., 2023a; Zhou et al., 2024). Despite these advances in understanding, the involvement of m^6^A in astrocyte-mediated neurovascular crosstalk remains unexplored, and represents a critical knowledge gap given the dual roles of astrocytes as vascular architects and BRB sentinels.

To investigate the unexplored role of m^6^A in retinal astrocytes, we used a mouse model with retinal astrocyte-specific conditional knockout of *Virma* (*Virma* conditional knockout [cKO] mice). Our findings revealed how m^6^A dysregulation in astrocytes impaired developmental clearance, ultimately causing retinal vessel regression and vision loss. These results provide mechanistic insights into the pathological changes in retinal astrocytes and vasculature that are observed in conditions such as non-proliferative diabetic retinopathy, familial exudative vitreoretinopathy, and retinopathy of prematurity. Moreover, this work identifies VIRMA as a potential therapeutic target for vascular-associated retinopathies and establishes m^6^A-mediated post-transcriptional regulation as a critical mechanism of neurovascular homeostasis.

## Methods

### Animals

The *Gfap*-Cre mice (B6.Cg-Tg (*Gfap*-cre) 73.12Mvs/J, RRID: IMSR_JAX:012886) had a C57BL/6J genomic background. The Ai14 mice (B6; 129S6-*Gt(ROSA)26Sor*^tm14(CAG-tdTomato)Hze^/J, RRID: IMSR_JAX: 007908) originated from 129S6/SvEvTac and C57BL/6NCrl strains. The *Virma*^flox/flox^ mice were constructed by Bin Shen Laboratory (China, Nanjing) with a C57BL/6N genomic background. *Virma*^flox/flox^ mice were crossed with *Gfap*-Cre mice to produce astrocyte-specific *Virma* knockout in *Gfap*-Cre;*Virma*^flox/flox^ mice (designated *Virma* cKO mice), and all littermate control animals were *Virma*^flox/flox^ mice in the following experiments. Ai14 (floxed) mice were crossed with *Gfap*-Cre mice to produce the first filial generation for labeling glial fibrillary acidic protein (GFAP)-expressing cells with tdTomato fluorescent protein. Animals of both sexes were included in our study unless specified in the experimental methods. All animals were housed in a specific pathogen–free animal facility and maintained under a 12/12 hour light/dark cycle with lights on at 08:00 a.m. The temperature was maintained at 21 ± 1°C, and humidity at 50% ± 5%, with free access to water and food (license No. SYXK (Yue) 2021-0119). Animal experiments were performed under protocols approved by the Institutional Animal Care and Use Committee of Shenzhen Institute of Advanced Technology, Chinese Academy of Science (approval No. SIAT-IACUC-190305-NS-ZT-A0695, March 11, 2019).

### Genotyping

Toes from the mice were clipped, and these samples were then incubated with 50 µL of lysis buffer (1× phosphate-buffered saline [PBS], 0.1% Triton X-100, 100 µg/mL proteinase K [Invitrogen, Carlsbad, CA, USA, Cat# 25530049]) at 55°C for 3 hours, and then at 98°C for 10 minutes. The supernatants were used for polymerase chain reaction (PCR) verification (2× Hieff® PCR Master Mix; Yeasen, Shanghai, China, Cat# 10102ES08) with the following primers: *Gfap*-Cre-(WT), forward: CTA GGC CAC AGA ATT GAA AGA TCT; *Gfap*-Cre-(WT), reverse GTA GGT GGA AAT TCT AGC ATC ATC C; *Gfap*-Cre-(knock-in), forward: TCC ATA AAG GCC CTG ACA TC; *Gfap*-Cre-(knock-in), reverse: TGC GAA CCT CAT CAC TCG T; *Virma*, forward1: TGC CAC ATA GAT GCG ATG CC; *Virma*, reverse1: CAA GAC CCT GGG TTG AAC CC; *Virma*, reverse2: AGC TCA GAC CAT AAC TTC GT.

### Immunofluorescent staining

For immunofluorescence of frozen slices of eyeballs or brains from mice of different ages, the animals were first perfused with PBS and subsequently with 4% paraformaldehyde (PFA) in PBS. The brain and bilateral eyeballs were then removed and fixed in 4% PFA overnight at 4°C, followed by immersion in 30% sucrose in PBS for 1 day, and immersion in 30% sucrose in PBS for another day. The dehydrated tissue was then immediately frozen and stored at –80°C. The eyeballs and brain tissue were sliced at 12 µm and 35 µm, respectively, using a freezing microtome (CM1950, Leica, Wetzlar, Germany). For immunofluorescence of flat-mounted retinas (Kautzman et al., 2018; Rattner et al., 2019), mice were perfused and fixed in 4% PFA for 1 hour at room temperature. The dissected retinas from the eyeballs were then rinsed twice with PBS.

The immunofluorescence procedures were performed according to a previous study with minor modifications (Shi et al., 2018). Frozen sections or whole-mount retinas were incubated with 0.3% Triton X-100 in PBS for 15 minutes and then rinsed three times with PBS. The tissue was then incubated with a blocking reagent (1× PBS, 0.3% Triton X-100, 5% bovine serum albumin) for 2 hours at room temperature (25°C), primary antibody solution overnight at 4°C in a dark place, and secondary antibody solution for 2 hours at room temperature, followed by the final incubation of Hoechst 33342 for 10 minutes at room temperature. Primary and secondary antibodies were diluted in an antibody solution (1× PBS, 0.3% Triton X-100, 1% bovine serum albumin). The primary antibodies used were: mouse anti-GFAP (1:600, Sigma, St. Louis, MO, USA, Cat# G3893, RRID: AB_477010), rabbit anti-GFAP (1:1000, Abcam, Cambridge, UK, Cat# ab7260, RRID: AB_305808), rabbit anti-VIRMA (1:100, Cell Signaling Technology, Danvers, MA, USA, Cat# 88358S), goat anti-transcription factor Sox9 (1:1000, R&D Systems, Minneapolis, MN, USA, Cat# AF3075-SP), rabbit anti-Ki67 (1:500, Abcam, Cat# ab16667, RRID: AB_302459), rabbit anti-cleaved Caspase3 (1:400, Cell Signaling Technology, Cat# 9661T, RRID: AB_2341188), rabbit anti-glutamine synthetase (GS; 1:2000, Sigma, Cat# G2781, RRID: AB_259853), rabbit anti-β3-tubulin (1:1000, Abcam, Cat# ab18207, RRID: AB_444319), anti-GS-IB4 isolectin (1:100, Invitrogen, Cat# I21414), mouse anti-glutamine synthetase (GS; 1:2500, BD Biosciences, Franklin Lakes, NJ, USA; Cat# 610517, RRID: AB_2313837), and rabbit anti-ionized calcium binding adaptor molecule 1 (Iba1; 1:1000, Wako Chemical, Osaka, Japan, Cat# 01919741, RRID: AB_839504). The secondary antibodies used were: Alexa Fluor 594 (goat anti-mouse, 1:500, Invitrogen, Cat# A11005, RRID: AB_2534073; donkey anti-rabbit, 1:200, Yeasen, Cat# 34212ES60, RRID: AB_2920875), Alexa Fluor 488 (goat anti-rabbit, 1:500, Invitrogen, Cat# A11008, RRID: AB_143165; donkey anti-goat, 1:500, Invitrogen, Cat# A11055, RRID: AB_2534102), DyLight™ 405 (donkey anti-rabbit, 1:200, Yeasen, Cat# 34204ES60, RRID: AB_3676094), and Cy2-conjugated streptavidin (Jackson ImmunoResearch, West Grove, PA, USA, 1:500, Cat# 016220084, RRID: AB_2337246). A terminal deoxynucleotidyl transferase dUTP nick end labeling (TUNEL) assay was conducted to detect apoptotic cells using the DeadEnd™ Fluorometric TUNEL System (Promega, Madison, WI, USA, Cat# G3250) according to the manufacturer’s protocol.

Fluorescence images were obtained using a virtual slide scanner (VS120, Olympus, Tokyo, Japan) or confocal microscope (LSM900, Zeiss, Oberkochen, Germany). Three to four fields per mouse from the central or peripheral region of flat-mount retinas were analyzed using ImageJ/FIJI software (version: 1.53q, National Institutes of Health, Bethesda, MD, USA) to measure the number of immunofluorescence-positive cells. All fields taken for each mouse were averaged or each field served as an *n* value.

### Western blot analysis

We extracted mouse tissue, including the retina, eyeball, hippocampus, and cortex, from an adult male mouse. The tissue was digested and homogenized in lysis buffer consisting of 0.1% phosphatase inhibitor (MCE, Shanghai, China, Cat# K0021), 1% proteinase inhibitor (MCE, Cat# K0010), and radioimmunoprecipitation assay lysis buffer (MCE, Cat# K1001). We determined the protein concentration of the samples using a bicinchoninic acid protein assay kit (Invitrogen, Cat# 23227) and loaded 32 µg protein per sample into a 10% sodium dodecyl sulfate (SDS) polyacrylamide gel electrophoresis gel. The protein was then transferred onto nitrocellulose membranes and blocked with 5% skim milk blocking solution for 2 hours at room temperature. We then incubated the membranes in primary antibody solution overnight at 4°C and in secondary antibody solution for 2 hours at room temperature. Next, we briefly incubated the membranes with a chemiluminescence reagent (PerkinElmer, Waltham, MA, USA, Cat# NEL104001EA) and detected the signals using a ChemiDoc (Bio-Rad, Hercules, CA, USA).

The primary antibodies used were rabbit anti-VIRMA (1:1500, Proteintech, Chicago, IL, USA, Cat# 25712-1-AP, RRID: AB_2880204), mouse anti-CRE (1:1000, Millipore, Burlington, MA, USA, Cat# MAB3120, RRID: AB_2085748), and mouse anti-glyceraldehyde 3-phosphate dehydrogenase (GAPDH; 1:5000, Abcam, Cat# Ab8245, RRID: AB_2107448). The secondary antibodies used were horseradish peroxidase–conjugated Affinipure immunoglobulin (Ig)G (goat anti-mouse, 1:2000, Proteintech, Cat# SA00001-1, RRID: AB_2722565; goat anti-rabbit, 1:2000, Proteintech, Cat# SA00001-2, RRID: AB_2722564).

### Quantitative reverse transcription-polymerase chain reaction

We extracted total RNA from the retina, hippocampus, or striatum using a standard RNA extraction and purification protocol using TRIzol® Reagent (Invitrogen, Cat# 15596018) according to the manufacturer’s recommended procedures. The total RNA was then reverse-transcribed into cDNA libraries using a cDNA synthesis kit (Vazyme, Nanjing, China, Cat# R212-02) according to the manufacturer’s instructions. Next, we performed quantitative reverse transcription-polymerase chain reaction (qRT-PCR) using Universal SYBR qPCR master mix (Vazyme, Cat# Q511-02) and detected the signals using Quant Studio3 (Applied Biosystems, Foster City, CA, USA). Each assay was performed in technical duplicates. The relative expression ratio of target mRNA was normalized to Gapdh or Actb expression using the 2^–ΔΔCt^ method. The specific primer sequences used for the detection of target genes are listed in **Table 1**.

**Table 1:** Primers used for qRT-PCR

Primer name	Primer sequence
qRT-PCR	
*Cre*	F: TTG GCA GAA CGA AAA CGC TG
	R: TCA GCT ACA CCA GAG ACG GA
*Virma-exon2*	F: CAA AGT TCT CAT ATA GAT GTG GTT C
	R: CCA TAT GCT CTG TTGT CAG GTA AG
*Gfap*	F: GAG AAC AAC CTG GCT GCG TA
	R: CGG AGT TCT CGA ACT TCC TCC
*GS*	F: ACC CCT GGT TTG GAA TGG AG
	R: GTG GCC GTC TGT TCC CAT AA
*Rbpms*	F: GCC GAG AAG GAG AAC ACC C
	R: ATG AGA GAA CCT TCA TAG CCC TTG
*Gapdh*	F: AAC GAC CCC TTC ATT GAC
	R: TCC ACG ACA TAC TCA GCA C
*β-actin*	F: CAG TTC GCC ATG GAT GAC GAT
	R: ATC TGG GTC ATC TTT TCA CGG TTG
m^6^A-IP-qPCR	
*Gluc*	F: CGA CAT TCC TGA GAT TCC TGG
	R: TTG AGC AGG TCA GAA CAC TG
*Cluc*	F: GCT TCA ACA TCA CCG TCA TTG
	R: CAC AGA GGC CAG AGA TCA TTC
*Vegfa*	F: TTC GGG AAC CAG ACC TCT CA
	R: GAC CCA AAG TGC TCC TCG AA
*Sema6a*	F: CAC CCC TTA GCA GAC TGA CA
	R: TCA CTC CAT TCA GTG CCA CG
*Gapdh*	F: AAC GAC CCC TTC ATT GAC
	R: TCC ACG ACA TAC TCA GCA C

F: Forward; Gapdh: glyceraldehyde 3-phosphate dehydrogenase; Gfap: glial fibrillary acidic protein; GS: glutamine synthetase; IP-qPCR: immunoprecipitation-quantitative polymerase chain reaction; qRT-PCR: quantitative reverse transcription—polymerase chain reaction; R: reverse; Vegf: vascular endothelial growth factor; VIRMA: virilizer homolog VIR-like m^6^A methyltransferase associated.

### Optical coherence tomography

Optical coherence tomography (OCT) was performed as previously described (Mao et al., 2015). The animals were anesthetized with intraperitoneal Pentobarbital sodium (80 mg/kg), and the pupils were dilated with one drop of tropicamide phenylephrine eye drops (1:1 mixture of 1% tropicamide and 1% phenylephrine). 2.5% methylcellulose was used to lubricate the eyes. The mice were positioned in a stereotaxic frame, and the upper and lower eyelids were fixed with medical tape. Anterior segment OCT images were acquired by a near-infrared image OCT apparatus (TengMa Research Lab) with averaging 10 to 20 scans. The imaging device had a custom-made arm to direct the light path to the center of the pupil. The captured images were adjusted using Photoshop and formed a 3D image.

### Histology

Eyeballs removed from male mice were fixed in Formal-Acetic-Alcohol (Servicebio, G1109) for 24 hours at room temperature, followed by paraffin embedding and sectioning at 4 µm. The sections were stained with hematoxylin and eosin (HE) and photographed using a light microscope (Olympus, BX53F2) (Hosoi et al., 2018).

### Quantification of total retinal astrocytes

First, images of the whole retina were acquired using a virtual slide scanner (VS120, Olympus). The total area of each retina was measured using OlyVIA software (Olympus). Second, we sampled retinal astrocyte density from the central region of flat-mount retinas. Three to four 20× images per mouse were acquired for analysis using a confocal microscope (LSM900, Zeiss). The density of astrocytes in each image was manually quantified using ImageJ/FIJI software. The total number of astrocytes in each retina was estimated by multiplying the Sox9+ astrocyte density by the total area of the flat-mount retina.

### Modeling

To estimate the contribution of microglial phagocytosis to developmental astrocyte loss upon *Virma* ablation, a model based on previous studies (Puñal et al., 2019) to calculate the developmental death of retinal astrocytes was applied in our research. The model predicts the remaining number of cells (*N*_T_) at the end of the death process, considering the rate at which cells are dying and the initial cell count (*S*). The rate of death is determined by two parameters: (1) the fraction of the cells visibly dead or dying on a given day (*D*_n_) and (2) the duration a dead cell remains visible on day *n* (*V*_n_; also known as a clearance time). Assuming constant *D*_n_ and *V*_n_ across development days, the model simplifies to the equation:


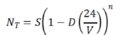


Given our parameters in cKO mice:

(*S*) represents the average number of retinal astrocytes at P6 (25804 cells),

(*N*_T_) denotes the average number of astrocytes at P12 (10561 cells),

(*n*) is 6 (P6 to P12 developmental span),

(*D*) is experimentally determined as 0.0093 (or 0.93%, the fraction of astrocytes fully enveloped by microglial processes from P6 to P12).

With these values, the clearance time (*V*) can be computed as:


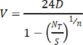


### Microglia–astrocyte interaction analysis

In the present study, we refined previous research methodologies (Puñal et al., 2019) to classify astrocyte–microglia interactions by analyzing three to four 40× images per mouse from the central or peripheral retinal area. Astrocytes were assessed based on their somata labeled with Sox9, and their interactions with microglial processes were categorized into four groups: touched, overlapped, partially enveloped, and fully enveloped. “Touched” indicated that the edges of the astrocyte somata were merely contacted by microglial processes, without encapsulation or overlapping. “Overlapped” referred to somata with partial overlap, with less than 50% of their circumference wrapped by microglial processes or with less than 50% co-localized area. “Partially enveloped” described somata with at least 50% of their circumference wrapped by microglial processes or with at least 50% co-localized area. “Fully enveloped” described somata completely surrounded by microglial processes. The percentage of four interactions per field was calculated by hand using FIJI and then averaged to one mouse (*n* value).

### Quantification of retinal vascular branch number and vessel density

For the statistical analysis of vascular branch number and vessel density, z-stacked confocal images were acquired to capture the GS-IB4^+^ signal of the three vascular plexuses. Using FIJI, vascular branch points were manually counted from four central fields per animal, each covering 0.41 mm^2^. A branch point was defined as any intersection of two blood vessels, following established protocols (Kautzman et al., 2018). Vessel density was determined by calculating the percentage of the total area of each field occupied by the blood vessel–covered area in FIJI, as per previously reported methods (Gnanaguru et al., 2023).

### Optomotor response assay

The optomotor response assay was conducted using a previously reported procedure (Niu et al., 2022). After overnight dark adaptation, mice were placed on a circular platform for adaptation of the optomotor system using 0.2 cycles per degree (cpd) with a Matlab program. Subsequently, spatial frequencies ranging from 0.025 to 0.4 cpd were tested, with 30 seconds of translation per direction. The left eye of the mouse was activated when the mouse’s head followed the grating clockwise (and *vice versa*) (Sergeeva et al., 2018). Visual acuity was determined by the maximum spatial frequency between the left and right eyes.

### Looming visual stimuli test

The looming visual stimuli test was performed as previously described (Zhou et al., 2019). In brief, mice were placed in an arena (40 cm × 40 cm × 35 cm) with a dark nest in the corner for shelter from visual stimuli. A computer monitor was positioned overhead for overhead visual stimuli. After at least 5 minutes of free exploration, the mice reached the center of the arena, and the looming stimulus was started. Within 300 ms, the visual angle of the black disc on the monitor expanded from 2 to 20 degrees, with this stimulus being repeated 15 times. The latency to return to the nest, length of stay in the shelter, and the freezing time were recorded and analyzed.

### Contextual and auditory fear conditioning

The fear conditioning test was performed as described previously (Shi et al., 2018). Training involved handling the mice for 3–5 minutes each day for 3 consecutive days. On the 4^th^ day, after exploring and habituating to the training chamber (flat roof with striped walls) for 3 minutes, the mice received three pairings of an auditory cue (78 dB, 2800 Hz, 28 seconds) with a foot shock (1.5 mA, 2 seconds). The chambers were cleaned with 75% alcohol to remove the odor after each test. After the last shock, mice remained in the training chamber for 1 minute. Testing was conducted 2 hours or 24 hours after training, during which the mice were placed back into the same training chamber for 3 minutes without any auditory cue or foot shock, and the freezing time was recorded for contextual fear memory tests using the JLBehv-FCSM-4 (JiLiang, Shanghai, China). For auditory fear memory tests, the mice were placed in a changed chamber (angular roof with white walls) to explore for 3 minutes, then received 3 pairings of an auditory cue (78 dB, 2800 Hz, 30 seconds) without footshock. After the auditory cue, mice remained in the changed chamber for 1 minute.

### Open field test

The control/cKO mice were handled for 3–5 minutes each day for three consecutive days before being placed in the center of a square open arena (40 cm width × 40 cm length × 40 cm height) on the fourth day. They were allowed to explore freely for 20 minutes to assess their locomotion and spontaneous activity. The activities of mice were recorded and analyzed using JLBehv-LAM-4 (JiLiang). The surface of the arena was cleaned with 75% alcohol after each trial to remove any odor.

### Forced swimming test

In the forced swimming test, the control/cKO mice were placed in an open cylindrical container (12 cm in diameter, 25 cm in height) filled with water to a depth of 18 cm at 24 ± 1°C. The mice were forced to swim for 6 minutes while being recorded using JLBehv-FSM-4 (JiLiang). The immobility time during the final 4 minutes was analyzed. The mice were considered immobile when they stopped struggling and remained motionless while floating in the water, only making necessary movements to stay afloat. The water was replaced with clean water after each mouse finished the test.

### Elevated plus maze test

The elevated plus-maze consisted of one central square (6 cm width × 6 cm length), two open arms (6 cm width × 26 cm length), and two closed arms (6 cm width × 26 cm length × 14.5 cm height) in a cross-shaped structure positioned 50 cm above the ground. The control/cKO mice were placed at the central square facing the open arm and allowed to explore freely for 5 minutes. The activity tracks were recorded and analyzed using JLBehv-EPMM-4 (JiLiang). The surface of the maze was cleaned with 75% alcohol after each trial to remove any odor.

### Light-dark box transition test

The Light-Dark Box Transition Test was performed as previously described (Shi et al., 2018). The apparatus consisted of a white box (light compartment, 25 cm width × 30 cm length × 30 cm height) and a black box (dark compartment, 25 cm width × 20 cm length × 30 cm height) separated by a partition with a hole. The light compartment was illuminated with a 200 Lx light, while the dark compartment had no extra light (< 5 lx). The control/cKO mouse was placed in the center of the light compartment, facing away from the hole, and allowed to explore freely for 10 minutes. The activities were recorded and analyzed using the JLBehv-PAM-2 (JiLiang). The bottom of the box was cleaned with 75% alcohol to remove the odor after each trial.

### Single-nucleus RNA sequencing analysis

Single-nucleus RNA sequencing (snRNA-seq) analysis was performed on the retinas of adult male mice from the control group (*n* = 5) and *Virma* cKO group (*n* = 6). Retinal tissue was dissociated into a single-nucleus suspension using nuclear lysis buffer (0.1% NP-40, 10 mM Tris-HCl, 146 mM NaCl, 1 mM CaCl_2_, 21 mM MgCl_2_, and 40 U/mL RNase inhibitor) and washed with wash buffer (10 mM Tris-HCl, 146 mM NaCl, 1 mM CaCl_2_, 21 mM MgCl_2_, 0.01% bovine serum albumin, and 40 U/mL RNase inhibitor). Single-nucleus gel beads were generated in oil droplets using the Chromium Controller (10× Genomics, Pleasanton, CA, USA, GCG-SR-1), followed by library construction using the Chromium Single Cell 3′ Reagent Kit (version 3, 1000020) according to the manufacturer’s instructions. The libraries were sequenced using the Illumina NovaSeq 6000 platform (Illumina, San Diego, CA, USA) with paired-end reads of 150 bp.

For snRNA-seq data preprocessing, we followed a previously reported procedure (Yang et al., 2021). The Cell Ranger software pipeline (10x Genomics, version 3.1.0) was used to demultiplex cellular barcodes, map reads to the genome and transcriptome, and down-sample reads to generate normalized aggregate data across samples. The unique molecular identifier count matrix was processed using the R package Seurat (version 3.0) (Butler et al., 2018) to filter out low-quality cells with unique molecular identifiers/gene numbers outside the limit (mean value ± 2 standard deviations) and further remove low-quality cells in which a certain percentage of counts belonged to mitochondrial genes. Library size normalization was performed in Seurat on the filtered matrix to obtain the normalized count.

The top variable genes across single cells were identified using a previously reported method (Macosko et al., 2015). Briefly, after the average expression and dispersion were calculated for each gene, genes were placed into several bins. Cells were then clustered and visualized using uniform manifold approximation and projection based on the data from the principal component analysis.

Differentially expressed genes (DEGs) were identified using the Seurat package (Butler et al., 2018). *P*-value < 0.05 and |log2foldchange| > 0.58 (or |log2foldchange| > 0.26) was set as the threshold for significantly differential expression. Gene Ontology (GO) enrichment analysis of DEGs was performed using R based on the hypergeometric distribution.

### Cell–cell communication analysis

Intercellular ligand–receptor interaction analysis was performed using the R package CellChat (version 1.1.3) (Jin et al., 2021). First, the normalized expression matrix was imported, and the CellChat object was created using the createCellChat function, followed by preprocessing using the identifyOverExpressedGenes, identifyOverExpressedInteractions, and projectData functions with default parameters. Next, potential ligand–receptor interactions were calculated using the computeCommunProb, filterCommunication (min. cells = 10), and computeCommunProbPathway functions. Finally, the intercellular communication network was aggregated using the aggregateNet function.

### m^6^A dot blot

The Retina total RNA was isolated as described above and quantified using UV spectrophotometry. RNA samples were mixed 1:2 with incubation buffer (1 mL mix: 210 µL 37% formaldehyde solution, 657 µL formamide, 133 µL 10×MOPS), denatured for 5 minutes at 65°C. Drop 1 µL of RNA onto a Positively Charged Nylon membrane (Invitrogen, AM10100), and RNA was cross-linked to the membrane with UV light at 254 nm. After washing the membrane in Tris buffered saline with Tween-20 (TBST; 0.1% Tween-20 in 1×TBS), the membrane was incubated in blocking buffer (0.5% nonfat milk in TBST) for 2 hours. Rabbit anti-m^6^A antibody (Synaptic Systems, 202003) was diluted 1:1400 in blocking buffer and incubated on the membranes overnight. After 3 washes in 1×TBST followed by incubation of horseradish peroxidase-conjugated Affinipure IgG antibody (Goat anti-rabbit) for 2 hours at room temperature, the membrane was visualized using chemiluminescence reagent and then stained with 0.02% methylene blue for RNA loading control.

### m^6^A-immunoprecipitation-quantitative polymerase chain reaction

The retinas of adult control mice (*n* = 8–10, males) were combined into one sample. The extracted total RNA was quantified, and more than 30 µg of RNA per immunoprecipitation (IP) group (m^6^A and IgG groups) was used for IP. One-tenth this amount of RNA was removed as input and stored at –80°C before performing qPCR. The IP of m^6^A -modified full-length RNA was performed using an EpiMark *N*^6^-Methyladenosine Enrichment Kit (NEB, Ipswich, MA, USA, Cat# E1610S) with several modifications (Zeng et al., 2018). After adding 0.5 µL *Gluc* (m^6^A control RNA) and *Cluc* (unmodified control RNA) per sample, RNA was subjected to rotated incubation for more than 2 hours at 4°C with 1 µg m^6^A (Millipore, Cat# ABE572) antibody for m^6^A group or IgG (Millipore, Cat# PP64) antibody for IgG group in 450 µL binding buffer (50 mM Tris-HCl [pH 7.4], 150 mM NaCl, and 0.5% IGEPAL CA630). Next, 20 µL protein A/G beads per group were resuspended in the RNA–antibody mixture, followed by rotated incubation for 1.5 hours at 4°C. After washing twice with 1 mL high-salt buffer (50 mM Tris-HCl [pH 7.4], 1 M NaCl, 0.1% SDS, and 1% IGEPAL CA630) and three times with 1 mL binding buffer, RNA was released in the incubation of 200 µL proteinase K buffer (100 mM Tris-HCl [pH 7.4], 50 mM NaCl, 1 mM ethylenediaminetetraacetic acid, 0.2% SDS, 5% proteinase K) for 1 hour at 37°C. Eluted RNA was precipitated in a threefold volume of ethanol, a one-tenth volume of 3 M NaAC, and 1 µL glycogen, and was then transcribed to cDNA for qPCR. The IP efficiency was calculated using the following formula: ∆Ct = Ct_IP_ – Ct_input_ + log_2_10, %input = 2^–∆Ct^. The primer sequences of the detected genes are listed in **Table 1**.

### Statistical analysis

All data were evaluated using GraphPad Prism 9.4.1 (GraphPad Software, Boston, MA, USA, www.graphpad.com). Differences between two groups were analyzed using Mann‒Whitney *U* test in optomotor response assay, the comparison of differencesbetween two groups in other results were analyzed using a two-tailed unpaired *t*-test. Results are expressed as the mean ± standard error of the mean. Significance levels are indicated as *P* < 0.05.

## Results

### Disrupted ocular structure and diminished developmental elimination of retinal astrocytes in *Virma* conditional knockout mice

**Figure 1:**
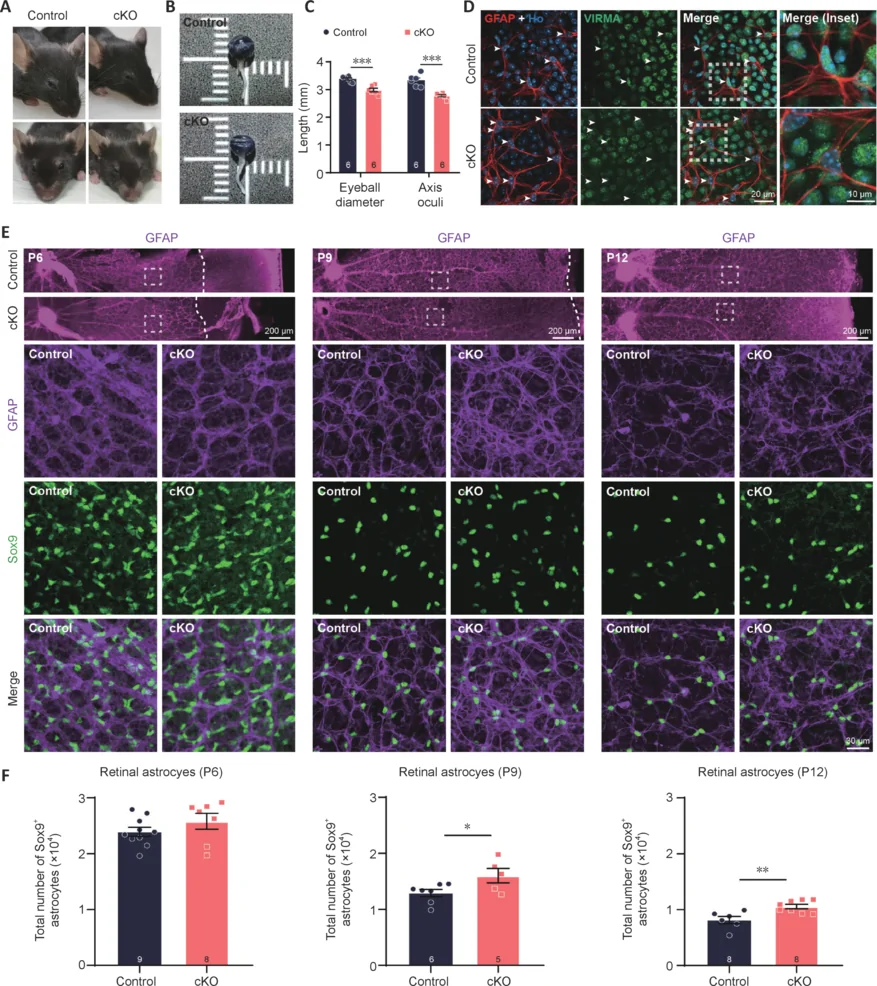
VIRMA deletion in retinal astrocytes causes ocular abnormalities and increases the number of developmental astrocytes. (A) Photographs of the side and front views of adult animals. Note the microphthalmia in the cKO mice. (B) The eyeballs of adult littermate control and *Virma*-cKO mice were prepared and photographed. (C) The eyeball diameter and axis oculi of eyeballs are plotted. (D) Representative pictures of flat-mounted retinal astrocytes from adult littermate control and *Virma* cKO mice immunostained with antibodies against GFAP and VIRMA. The white arrowheads indicate GFAP^+^ retinal astrocytes. Insets show single retinal astrocyte. Scale bar: 20 μm; scale bar inset: 10 μm. (E) Top panels, flat mounts of control and *Virma* cKO retinas at P6, P9 and P12 immunostained for the astrocyte network (GFAP). The white dashed lines mark the marginal area where astrocytes migrate in the retina. Scale bars: 200 μm. Bottom panels, high-magnification images of the central retina (white dashed box) showing retinal astrocytes (GFAP and Sox9). Scale bar: 30 μm. (F) The total number of retinal astrocytes at P6, P9 and P12 was estimated by multiplying the Sox9^+^ astrocyte density by the total area of the flat-mounted retina. Data are presented as the mean ± SEM. *P < 0.05, **P < 0.01, ***P < 0.001 (unpaired *t*-test). Numbers in bars: numbers of eyeballs (D, F). cKO: Conditional knockout; GFAP: glial fibrillary acidic protein; VIRMA/Virma: virilizer homolog VIR-like m^6^A methyltransferase associated.

**Figure 2:**
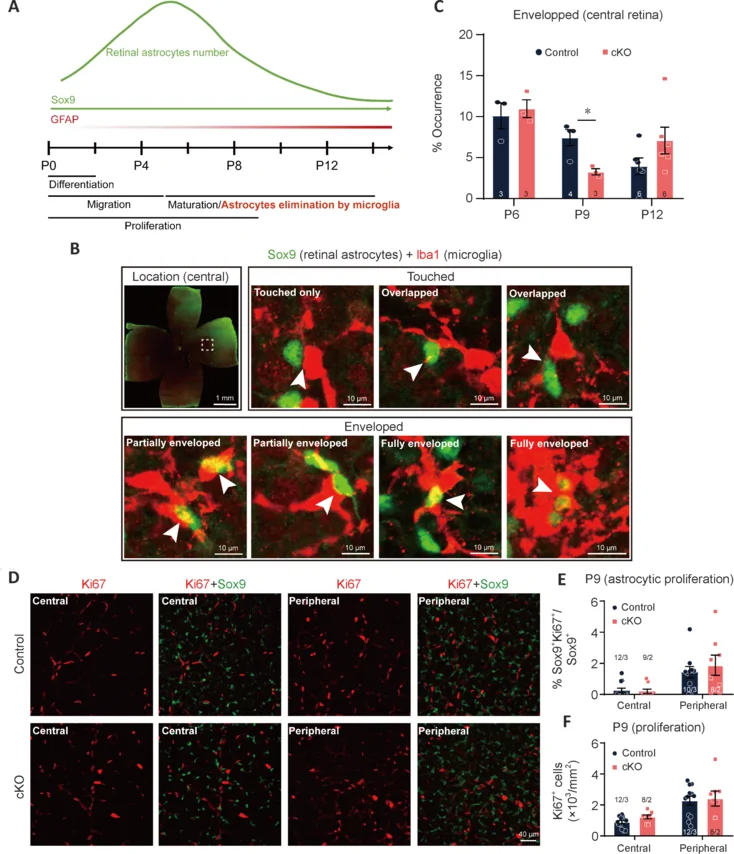
VIRMA deficiency in retinal astrocytes causes reduced elimination of astrocytes engulfed by microglia from P9. (A) Timeline of critical developmental events (top) and marker expression of retinal astrocytes (bottom). The expression of GFAP in astrocytes increases from the immature stage to the mature stage, and both immature and mature astrocytes are developmentally eliminated by retinal microglia during the death period from P5–P14. (B) The white dashed box indicates the image acquisition area of the central retina in the first picture. Scale bars: 1 mm. Examples of the four interaction categories are shown in other images and highlighted by arrows. If the soma of an astrocyte is contacted by a microglial process without wrapping and colocating, it is labeled “touched only.” When the colocalization area or circumference enveloped by a microglial process is less than or equal to 50%, the astrocyte somata are considered “overlapping,” whereas when the above indicators are greater than 50%, they are considered “partially enveloped.” An astrocyte soma is considered “fully enveloped” if the entire circumference of the soma is surrounded by a microglial process. Scale bars: 10 μm. (C) Astrocyte–microglia interactions quantified in control and *Virma* cKO central retinas stained with Sox9 and Iba1. A percentage of astrocyte somata touched (touched only and overlapped) and enveloped (partially and fully enveloped) by microglial processes at P6, P9 and P12 in the central retina. Besides, Additional Figure 4C shows a representative z-stack analysis of “fully enveloped” interactions. (D) Representative images of the central area (left two columns) and peripheral area (right two columns) of P9 flat-mounted retinas immunostained with Ki67 and Sox9. Scale bar: 40 μm. The number of proliferating retinal astrocytes (Sox9^+^ Ki67^+^) and total proliferating cells (Ki67^+^) at P9 is quantified in E and F, respectively. Data are presented as the mean ± SEM. *P < 0.05 (unpaired *t*-test). Numbers in bars: numbers of mice (C) and numbers of fields/numbers of mice (E, F). cKO: Conditional knockout; GFAP: glial fibrillary acidic protein; VIRMA/Virma: virilizer homolog VIR-like m^6^A methyltransferase associated.

We first evaluated the expression of the m^6^A writer VIRMA in the mouse brain and retina and demonstrated that the retina exhibited relatively high levels of VIRMA expression compared with the cortex and hippocampus of the brain (**Additional Figure 1A**). Immunostaining further confirmed high VIRMA levels in retinal and brain astrocytes (**Additional Figure 1B**), thus suggesting its potential astrocytic roles. To explore the physiological function of VIRMA, we generated astrocyte-specific *Virma* cKO mice by crossbreeding the *Virma*^flox/flox^ with *Gfap*-Cre mice (**Additional Figure 1C** and **D**) (Wei et al., 2015; Sun et al., 2017). Cre expression was validated via western blot, qPCR, and Ai14 reporter assays (**Additional Figure 1E–H**). Compared with control mice, cKO mice exhibited normal development with a slight decrease in body weight in male mice (**Additional Figure 1I**); however, they displayed pronounced microphthalmia and abnormal ocular structure (**Figure 1A–C** and **Additional Figure 1J–M**), indicating substantial defects in eye formation. Complete VIRMA ablation in adult retinal astrocytes was confirmed (**Figure 1D**), with knockout efficiency progressively increasing from 30.16% at postnatal day (P)3 to 95.12% by P15 (**Additional Figure 2A** and **B**), which was correlated with developmental GFAP upregulation (**Figure 2A**).

Next, we investigated whether VIRMA deletion in retinal astrocytes affects their developmental dynamics. Astrocyte precursor cells migrate from the brain to the retina along the optic nerve, subsequently spreading from the optic nerve head to the periphery of the retina. This migration is accompanied by the proliferation, differentiation, and maturation of retinal astrocytes. Because astrocyte precursor cell differentiation into early astrocytes precedes P0 (Chan-Ling et al., 2009)—that is, prior to efficient VIRMA knockout—we evaluated astrocyte migration via GFAP staining in P6/P9 flat-mounted retinas, and observed no significant differences between the control and cKO groups (**Figure 1E**). Under physiological conditions, the proliferation of retinal astrocytes peaks during the first postnatal week, followed by a sharp decrease as the result of elimination by microglial phagocytosis (Puñal et al., 2019). By P14, the astrocytes stabilize into a mature population (Chan-Ling et al., 2009; Puñal et al., 2019; Paisley and Kay, 2021). The quantification of astrocyte number using Sox9, a nuclear marker of astrocytes (O’Sullivan et al., 2017), revealed abnormal astrocytic accumulation in cKO retinas starting at P9 (1.4-fold increase), escalating to 2.1-fold by P35 (**Figure 1E**, **F**, and **Additional Figure 3A**, **B**), and persisting into adulthood (**Figure 1A**). Our analysis of astrocyte numbers throughout postnatal development was consistent with the findings of previous studies (Puñal et al., 2019; **Additional Figure 3B**) and indicates that VIRMA knockout may affect the developmental elimination of retinal astrocytes.

### *Virma* conditional knockout mice demonstrate reduced microglial phagocytosis of retinal astrocytes and disrupted retinal homeostasis

Previous studies have indicated that retinal microglia engulf retinal astrocytes during P5–14 to promote astrocyte removal (Puñal et al., 2019; Gnanaguru et al., 2023) (**Figure 2A**). Consequently, we investigated the interactions between retinal astrocytes and microglia in the central/peripheral regions of flat-mount retinas using Sox9/Iba1 co-staining (**Figure 2B** and **Additional Figure 4B**). On the basis of the contact between microglial processes and astrocytic somata, these interactions were categorized as: 1) touched (including touched only and overlapped) or 2) enveloped (partially/fully enveloped); the latter displayed phagocytic cup–like morphology (**Figure 2B**). Orthogonal views confirmed three-dimensional engulfment, excluding imaging artifacts (**Additional Figure 4C**). Notably, compared with the control groups in the central retina, we observed that more astrocyte soma are touched and contacted by microglial processes (**Additional Figure 4A**), while fewer astrocyte soma are enveloped and engulfed by microglial processes in cKO mice at P9, with no significant differences at P6 and P12 (**Figure 2C**). Similarly, astrocyte somata were more frequently touched by microglial processes in the cKO peripheral retina but there was no clear difference in the envelopment of astrocytes by microglia at P9 (**Additional Figure 4D** and **E**), potentially reflecting delayed GFAP expression and incomplete VIRMA knockout in immature peripheral astrocytes.

To elucidate the effects of microglia engulfment on developmental astrocytic clearance, we used a modeling approach based on previous studies (Puñal et al., 2019). When we calculated the clearance time of cKO astrocytes based on the average occurrence of “fully enveloped” events (**Additional Figure 4F**), the theoretical decline curve predicted by the model aligned with the actual astrocyte numbers and forecasted a cKO astrocyte clearance time of 1.62 hours (**Additional Figure 4G**), which exceeded that of prior reports (45–55 minutes) (Puñal et al., 2019). This delay likely results in the retention of more developmental astrocytes in the retina because of insufficient clearance by microglia.

To assess astrocyte proliferation, Ki67/Sox9 co-staining revealed comparable proliferative astrocytes and retinal cells in the central/peripheral regions of P9 cKO retinas versus control retinas (**Figure 2D–F**). Apoptosis assays (TUNEL/cleaved caspase-3) revealed minimal cell death with no differences between the two groups (**Additional Figure 4H** and **I**). Furthermore, to exclude the possibility that decreased astrocyte engulfment was caused by a reduced number of microglial cells in cKO mice, we quantified Iba1^+^ cell numbers and identified no differences in the central/peripheral retina at P9 (**Additional Figure 5C** and **D**). Collectively, these findings further suggest that VIRMA deficiency does not promote developmental astrocyte retention by modulating proliferation or apoptosis.

We next investigated whether elevated postnatal astrocytes affect other retinal cell types and disrupt retinal homeostasis. Intriguingly, GFAP expression was detected in some Müller glial cells of cKO mice that exhibited severe ocular defects (**Additional Figures 1K** and **5A**), indicating that these cells are activated in response to retinal injuries. However, Gs transcript levels were unchanged, suggesting that Müller glial numbers may not be affected in cKO retinas (**Additional Figure 5B**). Furthermore, microglial morphological abnormalities were observed in CKO retinas; these microglia were characterized by aberrant process extension into adjacent retinal layers (**Additional Figure 5E**). This altered spatial distribution suggests potential microglial responsiveness to disrupted retinal homeostasis (Karlstetter et al., 2010; Grigsby et al., 2014). Retinal ganglion cells, which were marked by β3-tubulin (Vecino et al., 2016), were reduced in cKO mice compared with the control group (**Additional Figure 5F** and **G**). These findings suggest that a disorganized astrocyte network during postnatal development contributes to dysregulation of the retinal microenvironment, triggering Müller glial and microglial responses and ganglion cell loss. In summary, VIRMA deletion in retinal astrocytes may impair developmental astrocyte removal via microglial phagocytosis, thereby potentially exacerbating retinal homeostasis disruption.

### Impaired retinal vascular integrity maintenance in *Virma* conditional knockout mice

**Figure 3:**
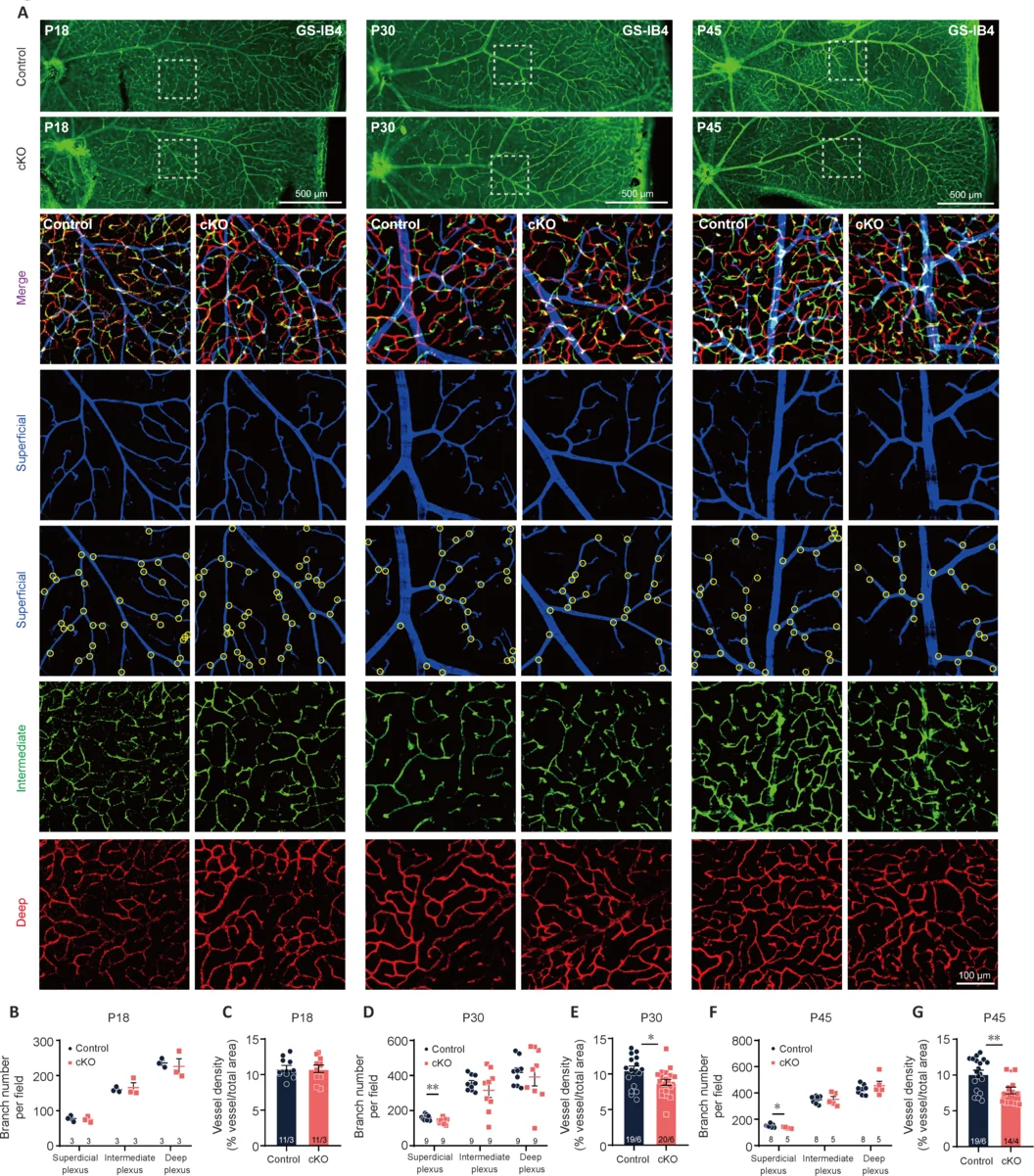
Impaired maintenance of the retinal vasculature in *Virma* cKO mice. (A) Top panels, flat mounts of control and *Virma* cKO retinas at P18, P30 and P45 immunostained for the retinal vasculature network (GS-IB4). Scale bars: 500 μm. Bottom panels, high-magnification images of the central retina (shown in Figure 2B) at various ages (P18, P30, P45) stained with GS-IB4. Scale bars: 100 μm. The GS-IB4 signal was pseudocolored by depth, with the superficial vascular plexus in blue, the intermediate vascular plexus in green, and the deep vascular plexus in red. The branches of the superficial vascular plexus are circled in yellow in the fourth row. Quantification of the number of vascular branches in the retina for each of the three vascular plexuses from mice at P18 (B), P30 (D), and P45 (F). Vessel density (%the area of GS-IB4^+^ fluorescence) of the superficial vascular plexus from mice at P18 (C), P30 (E), and P45 (G). (B–G) Data are presented as the mean ± SEM. *P < 0.05, **P < 0.01 (unpaired *t*-test). Numbers in the graph: numbers of mice (B, D, F) and numbers of fields/numbers of mice (C, E, G). cKO: conditional knockout; GS: glutamine synthetase; Virma: virilizer homolog VIR-like m^6^A methyltransferase associated.

Previous studies have demonstrated that retinal astrocytes play important roles in guiding the migration of vascular endothelial cells in the superficial vascular plexus by releasing VEGF (Stone et al., 1995; Dorrell et al., 2002; Rattner et al., 2019). The development of retinal vasculature occurs postnatally and is divided into three stages: P1–9 for the development of the superficial vascular plexus, P7–12 for the development of the deep vascular plexus, and P11–17 for the development of the intermediate vascular plexus (Wang et al., 2012). We therefore assessed the development and maturation of the three vascular plexuses at several time points, which were visualized using flat-mount GS-IB4 staining. We observed no detectable defects in the development and maturity of the superficial plexus at P6 and P9 (**Additional Figure 6A–C**) or of all three vascular plexuses at P18 (**Figure 3A–C**) in the cKO retinas. The migration or extension of vascular endothelial cells in cKO retinas appeared relatively similar to that of controls at P6 and P9 (**Additional Figure 6C**). This phenomenon might be explained by the finding that nearly half of the non-cKO astrocytes before P9 (**Additional Figure 2B**) were still able to support endothelial cell migration and angiogenesis for superficial vascular plexus formation. However, the deletion of VIRMA in retinal astrocytes led to a 13.5% reduction in vascular branches and a 16.2% decrease in vessel density of the superficial vascular plexus at P30 (**Figure 3A**, **D**, and **E**). Additionally, there was a 10.7% decrease in vascular branch number and a 22.8% decrease in vessel density of the superficial plexus at P45 (**Figure 3A**, **F**, and **G**). A similar defect in the superficial vascular plexus persisted into adulthood (**Additional Figure 6D** and **E**). These observations indicate that retinal vascularization remained unaffected in cKO animals before P18. However, the maintenance of retinal vasculature and vascular integrity were compromised from P30 onward, with phenotypes of abnormal vascular remodeling and vessel regression, suggesting a potential impairment of retinal homeostasis and indicating that these factors may contribute to visual function defects.

Before the maturation of retinal vasculature, the retina is nourished by the hyaloid vasculature, which is present in the vitreous (Edwards and Lefebvre, 2013). Retinal astrocytes are reportedly critical for the regression of hyaloid vasculature (Rattner et al., 2019). We therefore investigated whether the absence of VIRMA in retinal astrocytes affects this process. No detectable developmental or regression defects of the hyaloid vasculature were observed in the cKO group from P3 to P18 (**Additional Figure 6F**).

### Visual function impairment and behavioral analysis of *Virma* conditional knockout mice

**Figure 4:**
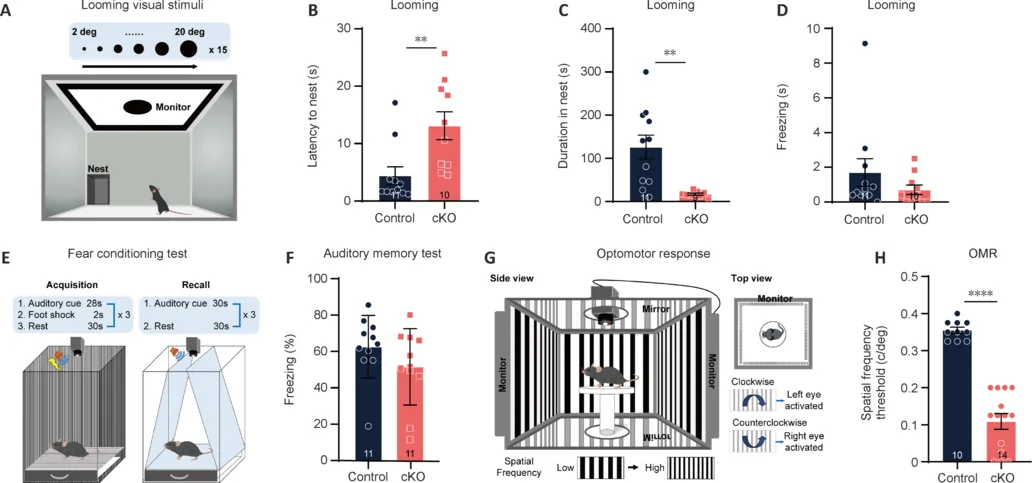
Impaired visual function in *Virma* cKO mice. (A) Schematic drawing of the looming animation and testing arena. (B–D) The latency of the mice to jump into the nest after the looming stimuli (B), duration in the nest (C), and freezing duration were recorded (D), *n* = 11 control *vs.* 10 cKO. (E) Schematic drawing of the fear conditioning test. (F) The proportion of freezing time was quantified in the tone-cued fear conditioning test. (G) Setup of the optomotor response (OMR) system. (H) The spatial frequency threshold (c/deg) was tested for visual acuity and compared between control and cKO mice. (B–D, F, H) The data are presented as the mean ± SEM. **P < 0.01, ****P < 0.0001 (unpaired Student’s *t*-test [B–D, F], Mann‒Whitney *U* test [H]). Numbers in bars: numbers of mice (B–D, F, H). cKO: Conditional knockout; Virma: virilizer homolog VIR-like m^6^A methyltransferase associated.

The observed retinal vascular maintenance and homeostasis impairment that resulted from astrocytic VIRMA deletion prompted us to investigate visual function in *Virma* cKO mice. We performed behavioral tests to assess vision-related responses. In the looming visual stimuli test (Yilmaz and Meister, 2013), mice were exposed to an expanding disc stimulus that mimics an approaching aerial predator in the upper visual field (**Figure 4A**). When the mimic predator (looming stimuli) appeared, the control mice quickly fled to the safety nest and stayed there, whereas the cKO mice showed no obvious flight response (**Figure 4B–D**), suggesting visual impairment or an altered fear response. However, auditory fear conditioning tests confirmed intact fear learning and responses in the cKO mice (**Figure 4E** and **F**), although vision-dependent contextual fear responses were impaired (**Additional Figure 7A**). Further validation using the optomotor response system revealed severely reduced visual acuity in cKO mice (**Figure 4G** and **H**). Thus, the deletion of VIRMA in retinal astrocytes impairs visual function in mice.

Given the abundance of astrocytes in the brain, we wondered whether normal brain function, such as motor abilities and general emotional states, is affected by VIRMA deletion in GFAP^+^ cells. No abnormalities were observed in motor ability, anxiety-like behavior, or depressive-like behavior in the open field and forced swimming test (**Additional Figure 7B** and **C**). Although cKO mice showed increased open-arm exploration in the elevated plus maze (**Additional Figure 7D**), this likely reflected their poor visual ability to discriminate between the open and closed arm rather than reduced anxiety. Furthermore, the light–dark transition test confirmed normal anxiety levels in cKO mice (**Additional Figure 7E**). Collectively, these results indicate that VIRMA deletion in astrocytes severely compromises visual function without affecting motor abilities or general emotional states.

### Disrupted expression of m^6^A-modified genes related to cellular interaction and angiogenesis in the *Virma* conditional knockout mouse retina

**Figure 5:**
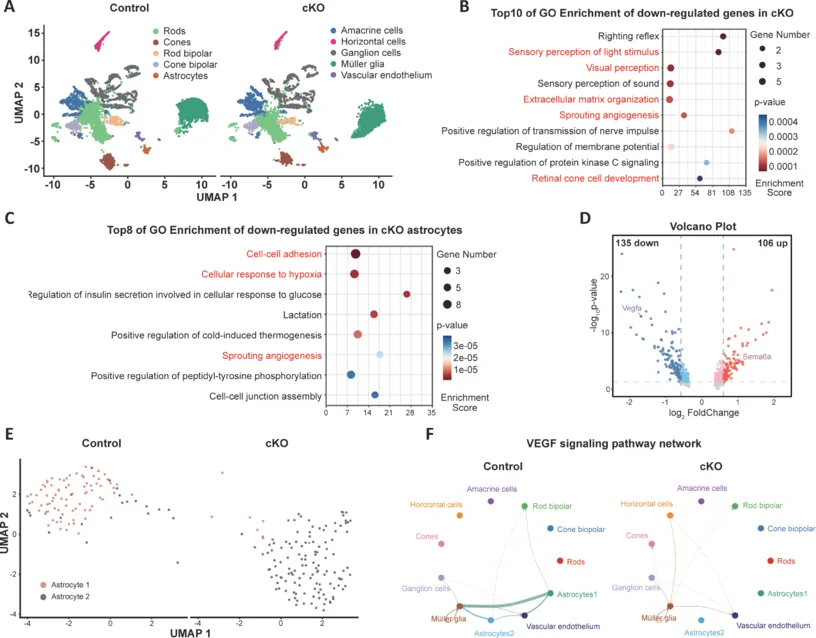
Single-nucleus RNA sequencing reveals the impaired function and interaction between retinal astrocytes and vascular endothelium cells in *Virma* cKO mice. (A) UMAP visualization of 6721 cKO mouse retina single-nuclei expression profiles (left) and 8261 control mouse retina single-nuclei expression profiles (right), clustered into 10 annotated cell types. (B) Bubble plot showing the top 10 most significantly enriched GO pathways in the biological process category of the downregulated genes (log_2_FC < –0.26, *P* value < 0.05) in the retinas of the cKO group. (C) The top 8 most significantly enriched GO pathways in the biological process category are shown in a bubble plot, which shows the differentially downregulated genes (log_2_ FC < –0.58, *P* value < 0.05) in cKO retinal astrocytes. (D) Volcano plot showing DEGs in retinal astrocytes between the cKO and control groups. The numbers of downregulated or upregulated genes in cKO retinal astrocytes are displayed (|log_2_FC| > 0.58, *P* value < 0.05). *Vegfa* and *Sema6a* are highlighted. (E) UMAP plot showing the clustering of 102 single nuclei from the control group (left) and 133 single nuclei from the cKO group (right) of retinal astrocytes into 2 subclusters, referred to as astrocyte types 1 and 2, respectively. (F) Circle plots showing the detected cell‒cell communication network for the VEGF signaling pathway. The arrow points to the recipient cell. Different node colors indicate different cell types. The thicker the line is, the stronger the interaction strength between two cell types. cKO: Conditional knockout; DEGs: differentially expressed genes; FC: fold change; GO: Gene Ontology; UMAP: uniform manifold approximation and projection; VEGF: vascular endothelial growth factor; Virma: virilizer homolog VIR-like m^6^A methyltransferase associated.

To explore the molecular mechanisms underlying the effects of *Virma* cKO in the retina, we performed snRNA-seq on retinas from cKO and control mice, because astrocytes represent a relatively minor fraction of the retina in terms of numbers. Following preprocessing and quality control (**Additional Figure 8A** and **B**), transcriptomic data from 14,982 single nuclei were categorized into 24 clusters using uniform manifold approximation and projection (**Additional Figure 9A**). The major cell types in rodent retinas were identified by annotating these clusters using specific marker genes (Gautam et al., 2021; Sun et al., 2021; Youkilis and Bassnett, 2021; **Additional Figure 9B** and **C**); 10 cell types were redefined and visualized (**Figure 5A**). GO analysis of the total downregulated DEGs revealed enrichment in biological process terms such as visual perception, extracellular matrix (ECM) organization, and sprouting angiogenesis, consistent with the observed visual and vascular defects of cKO mice (**Figure 5B**). A previous report noted that proper cellular interaction and ECM organization are imperative for microglia-mediated astrocyte engulfment (Gnanaguru et al., 2023). The downregulation of genes involved in this process may contribute to the reduced developmental elimination of retinal astrocytes and impaired retinal vascular integrity. A beeswarm plot identified astrocytes and vascular endothelial cells as the most affected cell types (**Additional Figure 9D**), indicating disrupted function and cellular interactions. Further GO analysis of DEGs in retinal astrocytes revealed downregulation in pathways such as cell–cell adhesion, cellular response to hypoxia, and sprouting angiogenesis, and wide enrichment in apoptotic mitochondrial changes with upregulated genes (**Figure 5C** and **Additional Figure 9E**), thus confirming functional impairments in cKO retinal astrocytes.

In the retina, the differentiation, proliferation, stabilization, growth, and survival of vascular endothelial cells are tightly regulated by a delicate balance between angiogenic and anti-angiogenic factors (Kota et al., 2012; Mrugacz et al., 2021). The volcano plot of astrocyte DEGs highlighted several potential target genes that are correlated with impaired vasculature, including the angiogenic factor VEGF (O’Sullivan et al., 2017; Apte et al., 2019; Rattner et al., 2019) and the anti-angiogenic factor semaphorin 6A (SEMA6A) (Dhanabal et al., 2005; Wei et al., 2015) (**Figure 5D**). On the basis of the gene expression profile obtained from snRNA-seq, the retinal astrocytes were clustered into two subtypes, designated astrocyte types 1 and 2 (**Figure 5E** and **Additional Figure 9F**). Notably, the distribution of these astrocyte subtypes in the cKO and control groups was markedly distinct. In the control retina, the primary type of astrocyte was type 1 (78.4%). By contrast, *Virma* inactivation in retinal astrocytes led to a significant shift from astrocyte type 1 to type 2 (94.7% of the cKO astrocytes are type 2), suggesting an aberrant transcriptional state in cKO retinas (**Additional Figure 9F**). To explore potential interactions between astrocyte subtypes and other retinal cells, we conducted CellChat analysis to investigate cellular communications. We observed robust interactions between astrocyte type 1 cells and other retinal cells in controls, thus highlighting the role of astrocytes type 1 in cellular communication. Conversely, the reduced proportion of type 1 astrocytes and diminished interactions suggest that impaired astrocyte communication occurs in cKO retinas (**Additional Figure 9G**). Among the vasculature-related signaling molecules and pathways that we focused on (**Additional Figure 9H**), we noted the disappearance of VEGF signaling from astrocytes in the cKO retina (**Figure 5F**) as well as increased SEMA6 signaling between astrocyte type 2 and vascular endothelial cells in cKO retinas (**Additional Figure 9I**). This disturbed balance between the two signaling pathways aligns with the severely impaired vasculature maintenance that was observed in the cKO retina.

**Figure 6:**
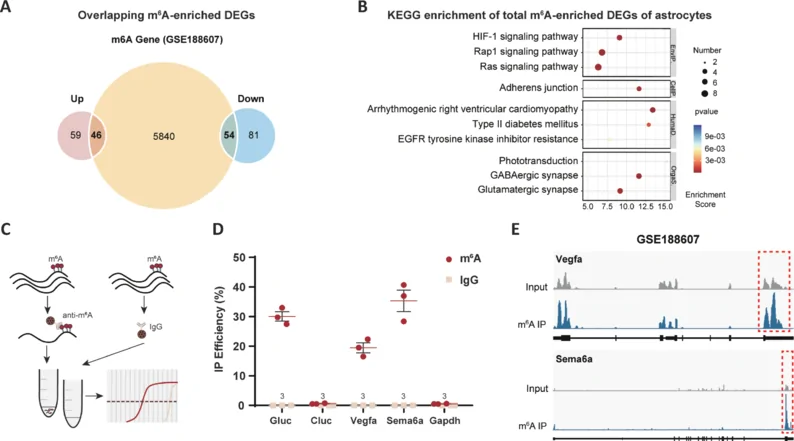
VIRMA regulates vascular remodeling and maintenance functions through m^6^A modifications of *Vegfa* and *Sema6a* transcripts in the retina. (A) Venn diagram showing the overlapping m^6^A-enriched DEGs of retinal astrocytes in the MeRIP dataset of GSE188607. Up- and downregulated DEGs were identified in cKO retinal astrocytes compared with control retinal astrocytes. (B) Bubble plot showing the top 3 most significantly enriched KEGG pathways among the total overlapping m^6^A-enriched DEGs of retinal astrocytes. (C) Schematic drawing of the m^6^A-IP-qPCR results. (D) The m^6^A levels of genes were quantified by m^6^A-IP. The results were normalized to the input RNA. Anti-IgG served as the negative control. Positive control: Gluc, the exogenous gene with m^6^A modification; negative control: Cluc, the exogenous gene without m^6^A modification; Gapdh, the known endogenous gene without m^6^A modification. (E) IGV views portraying the m^6^A peak distribution along transcripts of *Vegfa* and *Sema6a* revealed by MeRIP-seq (GSE188607). (D) Data are depicted as the mean ± SEM. Numbers in bars: numbers of samples. cKO: Conditional knockout; DEGs: differentially expressed genes; IP-qPCR: immunoprecipitation-quantitative polymerase chain reaction; KEGG: Kyoto Encyclopedia of Genes and Genomes; m^6^A: N^6^-methyladenosine; Vegf: vascular endothelial growth factor; VIRMA: virilizer homolog VIR-like m^6^A methyltransferase associated.

VIRMA represents the largest and most important structural component of the m^6^A methyltransferase complex, and promotes m^6^A deposition (Yue et al., 2018). Given that astrocytes constitute only 1%–2% of retinal cells (**Additional Figure 9C**), the application of m^6^A-seq in retinal astrocytes was markedly constrained. Instead, m^6^A dot blot assays revealed a moderate reduction in m^6^A levels in cKO retinas (**Additional Figure 10A** and **B**). When we compared DEGs in cKO astrocytes using an existing retinal methylated RNA IP sequencing dataset (Xin et al., 2022), we noted that 43.8% of upregulated genes and 40% of downregulated genes were m^6^A-modified, including *Vegfa*, *Sema6a*, and genes involved in ECM organization (**Figure 6A** and **Additional Figure 10C** and **D**). KEGG analysis of the total m^6^A-enriched DEGs in astrocytes highlighted enrichment in HIF-1 signaling and adherens junction pathway (**Figure 6B**), aligning with the functional changes that were observed in cKO astrocytes. The m^6^A modification of *Vegfa* and *Sema6* mRNAs was validated by conducting m^6^A-IP-qPCR while ensuring quality control using both exogenous (*Gluc* and *Cluc*) and endogenous (*Gapdh*) controls (**Figure 6C** and **D**). Furthermore, the distribution of m^6^A peaks in *Vegfa* and *Sema6a* transcripts was illustrated by analyzing the previously reported retinal methylated RNA IP sequencing dataset (Xin et al., 2022; **Figure 6E**). These findings suggest that the depletion of VIRMA may disrupt the expression of these target genes through reduced m^6^A modifications, subsequently leading to impaired astrocyte communication, developmental astrocyte removal, vascular integrity, and visual function in cKO mice.

## Discussion

Our study indicates that a loss of VIRMA in retinal astrocytes leads to abnormally diminished astrocyte removal through microglial phagocytosis, without affecting retinal vascularization in the early postnatal stage. However, mice with astrocyte-specific VIRMA ablation exhibited persistent impairment of vascular maintenance in the later postnatal stage, which further contributed to visual function impairment in adult mice. Mechanistically, single-nucleus transcriptomic analysis revealed that VIRMA loss resulted in disrupted expression levels of multiple m^6^A-modified genes that are associated with ECM organization and angiogenesis, which subsequently impaired intercellular communication between astrocytes and endothelial cells.

Multiple studies have highlighted the importance of m^6^A modifications for the appropriate temporal progression of gliogenesis in the brain, encompassing astrocytes and oligodendrocytes (Ma et al., 2018; Chen et al., 2019; Wang et al., 2021), as well as in regulating neural progenitor-to-glia cell transition in the retina (Xin et al., 2022); however, its function in specific cell types such as astrocytes has not previously been explored. In the present study, we report for the first time that VIRMA-mediated m^6^A regulates the developmental elimination of retinal astrocytes through microglial phagocytosis. However, we must note that our snRNA-seq did not detect microglial populations, likely because of their low abundance and the inherent limitations of snRNA-seq in capturing rare cell types (Slyper et al., 2020; Thrupp et al., 2020). This technical limitation hindered our ability to fully elucidate the molecular mechanisms underlying m^6^A-dependent regulation of microglial-mediated astrocyte engulfment. Nonetheless, our data demonstrated the severe disruption of astrocytic communication with other retinal cell populations (potentially including undetected microglia) in *Virma*-knockout astrocytes, as evidenced by the dysregulation of ECM organization and cell–cell adhesion pathways. Furthermore, emerging evidence suggest that the complement system is involved in mediating retinal astroglial clearance (Gnanaguru et al., 2023), thus suggesting a potential mechanistic link. Our findings indicate that although microglia retain their capacity to recognize developmental astrocytes in cKO mice, they exhibit impaired phagocytic efficiency. We therefore hypothesize that VIRMA in retinal astrocytes primarily regulates genes associated with “eat me” signals rather than “find me” signals during postnatal development (Xiao et al., 2024). Further experiments should focus on identifying these putative genes and elucidating their potential interplay with complement system components.

Our snRNA-seq analysis of adult retinas also revealed that astrocytes and vascular endothelial cells exhibited the most pronounced transcriptomic alterations following astrocytic *Virma* knockout. Sequencing data demonstrated that *Virma*-deficient retinal astrocytes displayed substantial transcriptional dysregulation, potentially disrupting astrocyte–endothelial communication through the modulation of cell–cell adhesion, cellular response to hypoxia, and sprouting angiogenesis, according to the GO analysis. These perturbations likely contribute to impaired vascular remodeling and the compromised integrity of retinal vasculature. Notably, we identified *Vegfa* downregulation and *Sema6a* upregulation in cKO mice, with parallel detection of m^6^A deposition on these transcripts in retinal tissue. Although cell-type-specific m^6^A-seq following astrocyte isolation would be ideal, technical challenges posed by the low abundance of retinal astrocytes (1%–2% of retinal cells) necessitate the future optimization of purification methods as well as low-input m^6^A-IP-qPCR approaches for validation.

As an important posttranscriptional modification, m^6^A dynamically controls mRNA metabolism through its interaction with various reader proteins (Fu et al., 2014; Jiang et al., 2021). The divergent expression patterns of *Vegfa* and *Sema6a* following VIRMA deletion suggest these m^6^A-modified transcripts may be regulated by distinct reader proteins. For example, m^6^A-modified transcripts targeted by YTHDF2 become unstable because YTHDF2 recruits the C-C chemokine receptor type 4–nuclear receptor subfamily 4 group A member 2 deadenylase complex. By contrast, insulin-like growth factor 2 binding proteins (IGF2BPs) target m^6^A-marked transcripts and prevent their degradation. Previous studies in colorectal cancer and pancreatic ductal epithelial cells have demonstrated the IGF2BP2/3-mediated regulation of VEGF (Guan et al., 2022; Liu et al., 2022), thus suggesting conserved astrocytic mechanisms. We propose that retinal astrocytes use (1) IGF2BP2/3-mediated *Vegfa* stabilization via m^6^A recognition; and (2) YTHDF2-dependent *Sema6a* degradation. VIRMA knockout reduces m^6^A deposition, thereby destabilizing *Vegfa* while prolonging *Sema6a* persistence, which collectively drives cKO phenotypes. However, this model requires validation through reader–protein interaction analyses and transcript-specific stability assays.

The current study has some limitations that should be noted. First, because of technical constraints, we were unable to collect a sufficient number of retinal astrocytes, which prevented astrocyte-specific m^6^A-seq analysis. Second, our study only examined single-cell sequencing data from adult cKO mice. Further analysis at early postnatal stages is needed to better understand the mechanisms underlying the observed impairment in astrocyte clearance.

Taken together, our findings reveal an essential role for VIRMA-mediated m^6^A modification in retinal astrocyte function: maintaining proper developmental astrocyte elimination, vascular integrity, and retinal homeostasis. Importantly, emerging evidence has underscored the dual regulatory roles of m^6^A in retinal biology—promoting physiological angiogenesis in retinal/choroidal vasculature (Shan et al., 2020; Yao et al., 2020; Wang et al., 2023a) and protecting against diabetes-induced endothelial cell and pericyte dysfunction (Suo et al., 2022; Zhou et al., 2023)—thus positioning them as promising therapeutic targets for vascular retinopathies. We propose two potential intervention strategies: pharmacological targeting of the scaffolding domain of VIRMA or precision editing of astrocytic m^6^A landscapes using clustered regularly interspaced short palindromic repeats–catalytically inactive Cas13. Such approaches may offer novel therapeutic strategies to restore homeostatic interactions between retinal astrocyte and vascular compartments while preventing BRB breakdown, which is a hallmark of progressive retinal disorders. Furthermore, our cKO mice may serve as a novel animal model to explore degenerative vascular diseases.

## Additional files:

***Additional Figure 1:*** *Generation, validation and phenotyping of conditional knockout mice.*

Additional Figure 1 Generation, validation and phenotyping of conditional knockout mice.
(A) Immunoblotting images and quantification of relative VIRMA levels in the retina, eyeball, hippocampus (hip), and cortex of adult C57BL/6 mice via western blotting. GAPDH was used as the internal reference. (B) VIRMA protein expression in GFAP^+^ astrocytes in the retina and hippocampus of P3 C57BL/6 mice. The high-magnification images are representative of the insets in the low-magnification images. Scale bar: low-magnification images, 300 μm; high-magnification images, 50 μm. (C) Schematic representation of the conditional knockout strategy for *Gfap*-Cre; *Virma*^flox/flox^ (cKO) mice. *Gfap*-Cre mice were crossbred with mice carrying the *Virma* gene with LoxP-flanked exon 2. (D) Genomic DNA was isolated from the retinas of *Virma*^flox/flox^ (littermate control) and *Gfap*-Cre; *Virma*^flox/flox^ (cKO) plants. The *Virma* flox allele was amplified via the PCR primers F1 and R1, and recombination of the *Virma* flox allele was amplified via the PCR primers F1 and R2 in C. cKO mice presented DNA bands that were positive for *Gfap*-Cre and specific knockout (KO) sequences. (E) Western blot analysis of Cre protein levels in the eyeballs (n = 3 mice) and hippocampi (hip) (*n* = 2 control vs. 3 cKO mice) of control and cKO mice. β-Actin and GAPDH were used as internal controls. (F) Relative levels of *Cre* transcripts were assessed in P3 retinas via qRT-PCR and are presented relative to *Gapdh* transcripts. (G) GFAP-expressing cells underwent Cre recombination, leading to the expression of the tdTomato red fluorescent protein in P0, P3 and P6 *Gfap*-Cre; Ai14 (flox/+) mice. The arrows indicate the positions of GFAP-expressing retinal astrocytes migrating from the optic nerve head to the peripheral retina at different time points. (H) Quantification of full-length *Virma* transcripts in the adult retina and hippocampus (hip) via qRT-PCR via primers designed to amplify the second exon of *Virma*, which was cut exclusively by *Cre* in cKO mice. Gapdh was used as the internal reference. (I) Pictures showing the differences in the size of adult mice and line charts showing the weight progression of male (n = 3 control vs. 5 cKO) and female (n = 4 control vs. 5 cKO) mice in the two groups. (J) Optical coherence tomography scans of adult mouse eyes reveal significant ocular abnormalities. The upper arrowhead indicates the cornea, the medial arrowhead indicates the iris, and the bottom arrowhead indicates the retina. Scale bar, 500 μm. (n = 2 mice). (K) Representative images of mice exhibiting minor, medium, and severe eye defects compared with littermate controls. (L) A stacked horizontal bar chart showing the proportions of the five grades of ocular defects identified by the standard in Additional Table 1, including grades 1 to 5 from mild to severe levels (n = 42 cKO mice). (M) Hematoxylin-eosin staining of ocular (scale bar: 400 μm), lens, cornea and retina (scale bars: 100 μm) samples from adult animals. Data are presented as the mean ± SEM.; ^*^P < 0.05, ^****^P < 0.0001; unpaired Student’s *t*-test (F, H); numbers in bars; and numbers of mice (F, H). cKO: Conditional knockout; GAPDH/Gapdh: glyceraldehyde 3-phosphate dehydrogenase; GFAP/Gfap: glial fibrillary acidic protein; qRT-PCR: quantitative reverse transcription-polymerase chain reaction; VIRMA: VIR-like m^6^A methyltransferase associated.

**Additional Table 1:** The definition description of 5 defect levels

Defect Level	Description
1	**Both eyes** show **minor defects** characterized by slightly reduced length and width of upper and lower eyelids.
2	**One eye** shows **minor defects**, and **another** shows **medium defects** characterized by moderately reduced length and width between two eyelids.
3	**Both eyes** show **medium defects**.
4	**One eye** shows **medium defects**, and **another** shows **severe defects** characterized by greatly reduced length and width between two eyelids or excessive eye discharge.
5	**Both eyes** show **severe defects**.

***Additional Figure 2:*** *Virma cKO mice exhibit retinal structure disorders.*

Additional Figure 2 *Virma* cKO mice exhibit retinal structure disorders.
(A) Representative confocal images of VIRMA knockout efficiency after costaining with VIRMA, Hoechst 33342 (Ho) and Sox9. The dashed white line circles the retinal astrocytes in which VIRMA was effectively knocked out in the field. Scale bar: 10 μm. (B) Quantification of VIRMA knockout efficiency at P3 (230 astrocytes from 2 control mice, 335 astrocytes from 3 cKO mice), P9 (82 astrocytes from 2 control mice, 84 astrocytes from 2 cKO mice) and P15 (37 astrocytes from 3 control mice, 73 astrocytes from 3 cKO mice). cKO: Conditional knockout; VIRMA/Virma: VIR-like m^6^A methyltransferase associated.

***Additional Figure 3:*** *VIRMA deletion in retinal astrocytes increases the number of developmental astrocytes after P9.*

Additional Figure 3 VIRMA deletion in retinal astrocytes increases the number of developmental astrocytes after P9.
(A) Top panels, flat mounts of control and *Virma* cKO retinas at P15, P18 and P35 immunostained for the astrocyte network (GFAP). Scale bars: 200 μm. Bottom panels, high-magnification images of the central retina (white dashed box) showing retinal astrocytes (GFAP and Sox9). Scale bar: 30 μm. (B) Quantification of total astrocyte numbers across development via multiplication of the Sox9^+^ astrocyte density by the total area of the flat-mounted retina, using retinas stained for Sox9. Data are presented as the mean ± SEM. ^*^P < 0.05, ^**^P < 0.01, ^****^P < 0.0001, unpaired Student’s *t*-test. cKO: Conditional knockout; GFAP/Gfap: glial fibrillary acidic protein.

***Additional Figure 4:*** *VIRMA deficiency in retinal astrocytes has no effect on the apoptosis of retinal astrocytes.*

Additional Figure 4 VIRMA deficiency in retinal astrocytes has no effect on the apoptosis of retinal astrocytes.
(A) Percentage of astrocyte somata touched (touched only and overlapped) by microglial processes at P6, P9 and P12 in the central retina. (B) The white dashed box indicates the image acquisition area of the peripheral retina. Scale bar: 1 mm. (C) Representative z-stack analysis of astrocyte soma “fully enveloped” by microglia processed with XZ and YZ orthogonal views. Scale bar: 10 μm. Percentage of astrocyte somata touched (touched only and overlapped) (D) and enveloped (partially and fully enveloped) (E) by microglial processes at P6, P9 and P12 in the peripheral retina. (F) The percentage of astrocyte somata fully enveloped by microglial processes at P6, P9 and P12 in cKO mice was averaged as the death rate parameter for modeling. (G) Modeling estimates the clearance time parameter V of CKO retinal astrocytes and suggests that the modeling based on the rate of “fully enveloped” microglia is roughly consistent with the actual value. (H) Representative images of P9 flat-mounted retinas immunostained with Sox9, TUNEL and CC3. Scale bar, 50 μm. The Tunel^+^ cells and CC3^+^ cells at P9 were quantified in I. Data are presented as the mean ± SEM. ^*^P < 0.05, unpaired Student’s *t*-test. Numbers in bars, numbers of mice (A, D, E), and numbers of fields/numbers of mice (I). CC3: Cleaved Caspase-3; cKO: conditional knockout; TUNEL/Tunel: terminal deoxynucleotidyl transferase dUTP nick end labeling; VIRMA: VIR-like m^6^A methyltransferase associated.

***Additional Figure 5:*** *Other retinal cell types, including glial cells and neurons, are affected in Virma cKO mice*

Additional Figure 5 Other retinal cell types, including glial cells and neurons, are affected in *Virma* cKO mice.
(A) Retinal sections from adult mice were double-stained with anti-GFAP and anti-GS (Müller glia) antibodies. The white arrowheads indicate GFAP^+^ expression in Müller glial cells in a fraction of adult cKO mice, which presented severe visual defects compared with those in the control group. Scale bar: 50 μm. (B) The Gs mRNA level normalized to the β-actin mRNA level was detected in the adult retinas via quantitative reverse transcription-polymerase chain reaction. (C) Representative images of flat-mounted retinas from P9 mice immunostained with Iba1 (microglia). Scale bar: 30 μm. The Iba1^+^ microglia density at P9 was quantified in D. (E) Representative images of retinal sections from adult mice immunostained with Iba1 (microglia) and Hoechst 33342 (Ho). Scale bar: 50 μm. (F) Representative micrographs of flat-mounted retinas from adult mice stained with β3-tubulin (ganglion cells) and Hoechst 33342. Scale bar: 10 μm. The number of ganglion cells per field (1000 μm^2^) from 3 control and 4 cKO mice was counted in G. (B, D, G) Data are presented as the mean ± SEM. ^*^P < 0.05, unpaired Student’s *t*-test. Numbers in bars, numbers of mice (B, D), and numbers of fields/numbers of mice (G). cKO: Conditional knockout; GFAP/Gfap: glial fibrillary acidic protein; GS: glutamine synthetase; Iba1: ionized calcium binding adaptor molecule 1; VIRMA: VIR-like m^6^A methyltransferase associated.

***Additional Figure 6:*** *Conditional elimination of the Virma in retinal astrocytes has no effect on early postnatal retinal vasculature or hyaloid vasculature.*

Additional Figure 6 Conditional elimination of the *Virma* in retinal astrocytes has no effect on early postnatal retinal vasculature or hyaloid vasculature.
(A) Representative confocal images of flat-mounted retinas from P6 mice stained with GS-IB4 isolectin. Scale bar: 50 μm. (B) The number of vascular branches of the superficial plexus. (C) Flat-mounted retinas from control and cKO mice at P6 and P9 were immunostained for retinal endothelium cells (GS-IB4). Scale bar: 100 μm. (D) Top panels, flat mounts of control and *Virma* cKO retinas at P80 immunostained for the retinal vasculature network (GS-IB4). Scale bar: 200 μm. Bottom panels, high-magnification images of the central retina at P80 stained with GS-IB4. The GS-IB4 signal was pseudocolored by depth, with the superficial vascular plexus in blue, the intermediate vascular plexus in green, and the deep vascular plexus in red. The branches of the superficial vascular plexus are circled in yellow in the fourth row. Scale bar: 100 μm. (E) Quantification of the number of vascular branches in the retina for each of the three vascular plexuses at P80. (F) The hyaloid vasculature and retinal vasculature were stained with GS-IB4. The white arrowheads in the left 2 panels indicate hyaloid vessels near the lens, whereas developmental regression of the hyaloid vasculature was almost complete at P12. The distribution of the 3 plexuses of the retinal vasculature can be observed in the P12 and P18 retinal sections. Scale bar: 100 μm. Data are presented as the mean ± SEM. ^*^P < 0.05, unpaired Student’s *t*-test. The numbers in the graphs are the numbers of mice (B, E). cKO: Conditional knockout; Virma: VIR-like m^6^A methyltransferase associated.

***Additional Figure 7:*** *Virma cKO mice present visual defects in other paradigms that are dependent on visual function.*

Additional Figure 7 *Virma* cKO mice present visual defects in other paradigms that are dependent on visual function.
(A) Fear conditioning in the same and altered context: The proportion of freezing duration was calculated and compared between the two groups. (B) Open field test (OFT): The total distance moved, average velocity and duration in the central zone were recorded and compared between groups to reflect the level of anxiety-like behavior. (C) The immobility time in the forced swimming test (FST) was compared between groups to assess despair-like behaviors. (D) Elevated plus maze (EPM): The left graph shows the duration in the open and closed arms, and the right graph shows the number of entries into the open and closed arms between control and cKO mice. (E) Light–dark box (LDB): The shuttle number and duration in the light and dark boxes were quantified and compared between control and cKO mice. Data are presented as the mean ± SEM. ^*^P < 0.05, ^**^P < 0.01, unpaired Student’s *t*-test, numbers in bars, and numbers of mice (A-E). cKO: Conditional knockout; Virma: VIR-like m^6^A methyltransferase associated.

***Additional Figure 8:*** *Quality control indicators for single-nucleus RNA sequencing before and after data filtering.*

Additional Figure 8 Quality control indicators for single-nucleus RNA sequencing before and after data filtering.
The violin plots elucidated the number of genes (nFeature RNA), the number of UMI (named nCount RNA in the graph), the proportion of genes per UMI (log_10_ Genes per UMI), the proportion of mitochondrial UMI (Percent.mito), the proportion of erythrocyte genes (Percent. HB) per cell before (A) and after (B) quality control. The quality control thresholds were set as follows: nFeature > 200, nCount RNA > 1000, log_10_ Genes per UMI < 0.7, Percent.mito < 0.1, Percent. HB < 0.05. cKO: Conditional knockout; UMI: unique molecular identifiers.

***Additional Figure 9:*** *Single-nucleus RNA sequencing reveals impaired retinal function in Virma cKO mice.*

Additional Figure 9 Single-nucleus RNA sequencing reveals impaired retinal function in *Virma* cKO mice.
(A) Uniform Manifold Approximation and Projection (UMAP) plot of 14,982 single nuclei from the cKO and control groups, revealing 24 clusters. (B) Expression of known specific marker genes (columns) in 10 cell types (rows) in the dot plot. (C) The percentages of 10 annotated cell types were compared between groups and are shown in the stacked vertical bar chart. (D) Beeswarm plot displaying the differentially expressed genes (DEGs) (|log_2_ FC| > 0.58, *P* value < 0.05) of each annotated cell type (upregulated DEGs in red, downregulated DEGs in blue). (E) Gene Ontology (GO) analysis of upregulated genes (log_2_ FC > 0.58, *P* value < 0.05) in cKO retinal astrocytes. (F) The percentages of cells assigned to each subcluster of retinal astrocytes in the cKO and control groups are shown in the bar graphs. (G) Circle plots showing the interaction strength of cell.cell communication in the control and cKO groups. (H) Heatmap displaying the normalized expression of seven sprouting angiogenesis-related genes among retinal astrocyte types 1 and 2. *Vegfa*, *Hif-2α* (hypoxia-inducible factor 2α), *Ccbe1* (collagen and calcium binding EGF domains 1), *Dysf* (dysferlin), and *Cxcl2* (C-X-C motif chemokine ligand 12) are involved in the positive control of angiogenesis, whereas *Sema6a* and *Mef2c* (myocyte enhancer factor 2C) are involved in the negative regulation of angiogenesis in various tissues. (I) Circle plots displaying the detected cell.cell communication network for the SEMA6 signaling pathway. The node size represents the total number of interactions (H). Node color indicates different cell types. Line thickness represents interaction strength, and the arrow points to the recipient cell (H, I). cKO: Conditional knockout; Virma: VIR-like m^6^A methyltransferase associated.

***Additional Figure 10:*** *Altered expression of potential target genes likely depends on VIRMA-mediated m*^*6*^*A modification.*

Additional Figure 10 Altered expression of potential target genes likely depends on VIRMA-mediated m^6^A modification.
(A) m^6^A dot blot assays of retinas from postnatal retinas of the control and cKO groups. RNAs were diluted to 150 ng, 75 ng and 37.5 ng and then loaded in the same volume. MB stain was used as an internal reference. The relative m^6^A level was measured in B. (C) The percentages of m^6^A-modified and non-m^6^A-modified DEGs among the up- and downregulated DEGs in cKO retinal astrocytes are shown in the stacked vertical bar chart. (D) According to the methylated RNA immunoprecipitation (MeRIP) dataset of GSE188607, the table indicates whether the m^6^A peak is present on the transcripts of the following genes: all DEGs of GO terms (extracellular matrix organization) in Figure 5B, *Vegfa* and *Sema6a*. -, no m^6^A peak; +, 1 or more m^6^A peaks. cKO: Conditional knockout; DEGs: differentially expressed genes; GO: Gene Ontology; MB: methylene blue; VIRMA: VIR-like m^6^A methyltransferase associated.

## Data Availability

*All relevant data are within the paper and its Additional files.*
